# Preoperative Total Serum Cholesterol and Patients' Survival in Resected Nonsmall Cell Lung Cancer

**DOI:** 10.1155/2012/463520

**Published:** 2012-11-26

**Authors:** Masaki Tomita, Takanori Ayabe, Tetsuya Shimizu, Kunihide Nakamura

**Affiliations:** Department of Surgery II, Faculty of Medicine, University of Miyazaki, Kihara 5200, Kiyotake, Miyazaki 889-1692, Japan

## Abstract

The association between hypocholesterolemia and lung cancer risk has been confirmed in some studies. The purpose of the study was to determine whether preoperative hypocholesterolemia (below normal range) is a prognostic factor for survival after nonsmall cell lung cancer (NSCLC) resection. Two hundred and sixty-two consecutive cases of resected NSCLC with a followup period for more than 5 years were reviewed retrospectively. In our results, there were only 13/262 patients having hypocholesterolemia. A significant association was observed between preoperative hypocholesterolemia and patients' survival. However, we failed to find the prognostic significance of preoperative hypocholesterolemia by univariate analysis. No statistical differences were also found by the comparison between 5-year survivors and the others. Our data indicates a trend toward an association between preoperative hypocholesterolemia and poorer survival in NSCLC; however, it did not reach statistical significance.

## 1. Introduction

It has been well accepted that hypercholesterolemia is a major risk factor for coronary heart disease. On the other hand, hypercholesterolemia has been associated with an increased risk of cancer mortality [1]. Some previous studies [2–5] observed that hypocholesterolemia was associated with a significantly increased risk of lung cancer.

To our knowledge, there is only one previous study that investigated the relationship between preoperative total serum cholesterol (TSC) and length of survival after nonsmall cell lung cancer (NSCLC) resection [6]. They reported that preoperative TSC might be an important prognostic factor for overall survival after NSCLC resection using the median value for TSC as the cutoff for dividing patients into low and high TSC groups [6]. However, they did not show the number of patients with TSC below normal range (hypocholesterolemia). We believe that hypocholesterolemia group should be defined as patients with TSC below normal range, not median value.

Therefore, in the present study, we investigated the prognostic significance of preoperative TSC, using the comparison between NSCLC patients with hypocholesterolemia and others.

## 2. Patients and Methods

The present retrospective study was conducted from 2001 through 2006 and included 262 patients with NSCLC who had underwent complete resection, which consisted of either a lobectomy or a pneumonectomy, together with regional lymph node dissection. Patients who did not have a preoperative dosage of cholesterol and who had a followup period of less than 5 years were excluded. The overall followup periods ranged from 62.7 to 132.5 months. Preoperative TSC was determined as part of routine preoperative examination; the normal range was 129–220 mg/mL (3.34–5.69 mmol/L). Pathological (p) tumor-node-metastasis (TNM) staging was recorded in all patients based on the 7th edition of the American Joint Committee on Cancer (AJCC)/International Union Against Cancer (UICC) classification. The baseline characteristics are summarized in Table 1. Followup information, including cause of death, was ascertained through a review of clinic notes and direct or family contact. Comparisons of categorical data between the two groups were made using Fisher's exact test with Yates' correction. Survival curves were obtained according to the Kaplan-Meier method. Comparison of survival curves was carried out using the log-rank test. Statistical calculations were conducted with JMP (SAS Institute Inc. Cary, NC, USA), and values of *P* less than 0.05 were accepted as being significant.

## 3. Results

There were only 13/262 (4.96%) patients having preoperative hypocholesterolemia. Although the number of patients with hypocholesterolemia was small, there were no significant differences between hypocholesterolemia group and others (Table 1). In addition, all 13 patients with hypocholesterolemia had not received any cholesterol-lowering therapies.

**Figure 1:**
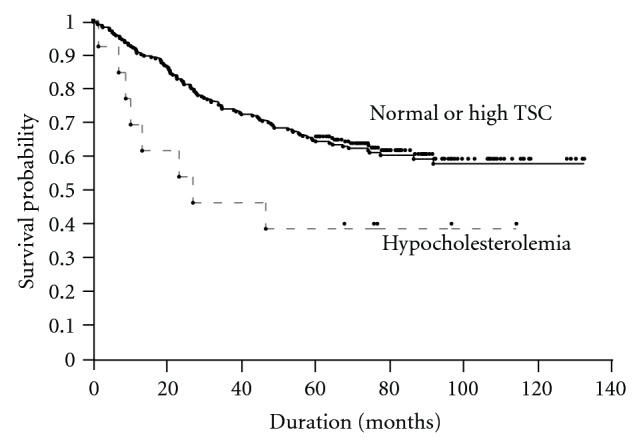
Survival of patients based on preoperative total serum cholesterol level. TSC: total serum cholesterol.

The survival curve based on preoperative total serum cholesterol level is shown in Figure 1. The 5-year survival rate was 38.46% among patients with preoperative hypocholesterolemia and 64.26% among others (*P* = 0.0289).

Univariate Cox proportional hazard regression analysis revealed that the gender (male versus female), the histological subtype (adenocarcinoma versus others), pT status (pT1 versus pT2-3), pN status (pN0 versus pN1-2), and serum CEA level (normal versus high) were related to patients' prognoses (Table 2). However, the prognostic significance of preoperative hypocholesterolemia did not reach statistical significance (*P* = 0.0557).

Besides prognostic analysis on overall survival of preoperative hypocholesterolemia, we compared the 5-year survival rate according to various potentially prognostic variables. As shown in Table 3, the five-year survivor group was characterized by significantly higher proportions of females, adenocarcinomas, pT1 status, pN0 status, and normal serum CEA level in comparison with those who did not survive 5 years. Among the patients with hypocholesterolemia, the number of 5-year survivors was smaller than that of nonsurvivors (5 versus 8). However, it did not reach statistical significance.

## 4. Discussion

Previous studies showed that hypercholesterolemia might increase lung cancer risk [2–5]. In addition, Sok et al. reported that preoperative TSC is a prognostic factor after NSCLC resection [6]. The association between hypocholesterolemia and cancer risk and poor prognosis is probably complex and largely unclear; however, some possible explanations exist. Muldoon et al. reported that hypocholesterolemic men had significantly fewer circulating lymphocytes, fewer total T cells, and fewer CD8+ cells than those with hypercholesterolemia [7]. Therefore, hypocholesterolemia might act by impairing the function of the immune system, thereby making defense mechanisms against tumor spread inadequate. It is also reported that cholesterol enhances the antigen-presenting function of monocytes [8]. Further, several pathways that are important in carcinogenesis, such as the sonic hedgehog and Akt pathways, are cholesterol sensitive [9]. Moreover, signal transducer and activator of transcription-6 (STAT6) is a member of the STAT family of latent transcription factor, and STAT6 knockdown is associated with inhibiting proliferation and enhancing apoptosis [10]. Dubey et al. found an inverse relationship of cholesterol biosynthesis and STAT6 in lung cancer cell lines [11]. 

Our data indicates a trend toward an association between preoperative hypocholesterolemia and patient survival in NSCLC; however, it did not reach statistical significance by univariate analysis and the comparison between 5-year survivors and the others. One of the most plausible reason for not reaching statistical significance is the small number of patients with hypocholesterolemia (4.96%). Sok et al. [6] used the median value for TSC as the cutoff for dividing patients into low and high TSC groups. However, we believe that hypocholesterolemia should be defined as patients with TSC below normal range, not median value. Since high intake of saturated fat or meat is known to elevate the TSC concentration [12], hypocholesterolemia might reflect the low nutritional status of patients. All patients in our study are surgical cases; therefore, it is possible that patients with low nutritional level might be excluded before surgery.

## 5. Conclusions

We showed a trend toward an association between preoperative hypocholesterolemia and poorer survival in NSCLC; however, it did not reach statistical significance. Further prospective studies using more large population in this area are warranted.

## Figures and Tables

**Table 1:** Clinicopathologic characteristics of study participants.

	Hypocholesterolemia	Others	*P* value
Age			
>65	6	94	0.753
≤65	7	155	
Gender			
Male	7	162	0.599
Female	6	87	
Histology			
Adenocarcinoma	10	180	0.963
Others	3	69	
p Stage			
I	9	170	0.815
II-III	4	79	
pT status			
pT1	4	141	0.123
pT2-4	9	108	
pN status			
pN0	9	189	0.830
pN1-2	4	60	
CEA			
Normal	6	151	0.454
High	7	98	

CEA: carcinoembryonic antigen.

**Table 2:** Results of univariate analysis.

	Favorable	Unfavorable	Risk ratio	95% CI	*P* value
Age	>65	≤65	0.7315	0.481–1.091	0.1270
Gender	Female	Male	0.4668	0.292–0.720	0.0004
Histology	Adenocarcinoma	Others	0.3858	0.263–0.572	<0.0001
pT status	pT1	pT2-3	0.3090	0.204–0.459	<0.0001
pN status	pN0	pN1-2	0.2882	0.196–0.427	<0.0001
serum CEA	Normal	High	0.5306	0.361–0.778	0.0012
TSC	Normal/high	Low	0.4565	0.237–1.021	0.0557

CI: Confidence interval, CEA: carcinoembryonic antigen, and TSC: total serum cholesterol.

**Table 3:** Comparison between 5-year survivors and the others.

	5-year survivor	Nonsurvivor	*P* value
Age			
>65	68	32	0.234
≤65	97	65	
Gender			
Male	95	74	0.003
Female	70	23	
Histology			
Adenocarcinoma	136	54	<0.001
Others	29	43	
pT status			
pT1	112	33	<0.001
pT2-4	53	64	
pN status			
pN0	143	55	<0.001
pN1-2	22	42	
CEA			
Normal	112	45	<0.001
High	53	52	
TSC			
Low	5	8	0.113
Normal/high	160	89	

CI: Confidence interval, CEA: carcinoembryonic antigen, and TSC: total serum cholesterol.
